# Auditory Gap-in-Noise Detection Behavior in Ferrets and Humans

**DOI:** 10.1037/bne0000065

**Published:** 2015-06-08

**Authors:** Joshua R. Gold, Fernando R. Nodal, Fabian Peters, Andrew J. King, Victoria M. Bajo

**Affiliations:** 1 Department of Physiology, Anatomy, and Genetics, University of Oxford

**Keywords:** ferret, human, behavior, temporal processing, auditory gap detection

## Abstract

The precise encoding of temporal features of auditory stimuli by the mammalian auditory system is critical to the perception of biologically important sounds, including vocalizations, speech, and music. In this study, auditory gap-detection behavior was evaluated in adult pigmented ferrets (*Mustelid putorius furo*) using bandpassed stimuli designed to widely sample the ferret’s behavioral and physiological audiogram. Animals were tested under positive operant conditioning, with psychometric functions constructed in response to gap-in-noise lengths ranging from 3 to 270 ms. Using a modified version of this gap-detection task, with the same stimulus frequency parameters, we also tested a cohort of normal-hearing human subjects. Gap-detection thresholds were computed from psychometric curves transformed according to signal detection theory, revealing that for both ferrets and humans, detection sensitivity was worse for silent gaps embedded within low-frequency noise compared with high-frequency or broadband stimuli. Additional psychometric function analysis of ferret behavior indicated effects of stimulus spectral content on aspects of behavioral performance related to decision-making processes, with animals displaying improved sensitivity for broadband gap-in-noise detection. Reaction times derived from unconditioned head-orienting data and the time from stimulus onset to reward spout activation varied with the stimulus frequency content and gap length, as well as the approach-to-target choice and reward location. The present study represents a comprehensive evaluation of gap-detection behavior in ferrets, while similarities in performance with our human subjects confirm the use of the ferret as an appropriate model of temporal processing.

Ethologically relevant auditory stimuli are often highly complex, with the auditory system displaying sensitivity to information represented not only by the frequency content of sound but also by the temporal structure of stimuli such as human speech and a wide range of animal vocalizations (Greenberg, Ainsworth, & Fay, 2004; Kuhl & Miller, 1978; Lisker & Abramson, 1964; Narins & Feng, 2006; Simmons & Fay, 2003; Sinnott & Adams, 1987; Viemeister, 2014). Gap-in-noise stimuli may be considered a useful experimental analogue of more complex acoustic features such as voice onset time and phonemic separation (Eggermont, 1999; Frisina, 2001; Phillips, Hall, & Boehnke, 2002; Tomita, Noreña, & Eggermont, 2004). As a simplified representation of these temporal variations in stimulus content, behavioral gap-detection paradigms have gained prominence as a means of interrogating central auditory temporal processing mechanisms and have been important in characterizing the effects of modified listening conditions, such as aging and hearing loss, on human temporal processing (Barsz, Ison, Snell, & Walton, 2002; Busby & Clark, 1999; Goŕdon-Salant & Fitzgibbons, 1993; Harris, Eckert, Ahlstrom, & Dubno, 2010; Schneider & Hamstra, 1999; Schneider, Pichora-Fuller, Kowalchuk, & Lamb, 1994; Snell & Frisina, 2000; Walton, 2010).

Across different experimental species, significant changes in gap-detection thresholds have been reported as sequelae of central or peripheral auditory insults, with particular emphasis placed recently on the possible utility of gap-detection measures as a means of indicating the presence of auditory perceptual abnormalities of a tinnitus-like nature (Engineer et al., 2011; Turner et al., 2006, Turner, Larsen, Hughes, Moechars, & Shore, 2012). Although the neural mechanisms underlying maladaptive changes in temporal processing remain to be elucidated, behavioral testing, when properly leveraged, may nevertheless allow for these mechanisms to be classified and understood (for review, see Gold & Bajo, 2014). The characterization of relevant animal models is thus critical to improving our understanding of both the neurobiological basis for these changes in perception and how the temporal structure of sounds is represented in the impaired human auditory system.

As a model experimental species, the ferret has emerged as particularly useful for investigating acoustically driven behaviors and for interrogating the underlying brain structures and physiological processes. In addition to vocalizing (Nodal & King, 2014) and hearing in a frequency range overlapping that of humans (Kelly, Kavanagh, & Dalton, 1986), ferrets have been used extensively in behavioral studies to investigate spatial hearing (Keating, Nodal, & King, 2014; King et al., 2007; Nodal, Bajo, Parsons, Schnupp, & King, 2008; Tolnai, Litovsky, & King, 2014), pitch processing (Walker, Schnupp, Hart-Schnupp, King, & Bizley, 2009; Yin, Fritz, & Shamma, 2010), and auditory scene analysis (Alves-Pinto, Sollini, & Sumner, 2012; Ma, Micheyl, Yin, Oxenham, & Shamma, 2010). Furthermore, the ferret’s well-described auditory cortical fields (Bizley, Nodal, Nelken, & King, 2005; Kelly, Judge, & Phillips, 1986; Nelken et al., 2004; Wallace & Harper, 1997; Wallace, Roeda, & Harper, 1997) and subcortical connectivity (Bajo, Nodal, Bizley, & King, 2010; Bajo, Nodal, Bizley, Moore, & King, 2007) have made this species well-suited for investigation of the neural circuitry responsible for adaptation to altered peripheral inputs (Bajo, Nodal, Moore, & King, 2010; Bizley, Nodal, Parsons, & King, 2007; Hartley et al., 2010; Irving, Moore, Liberman, & Sumner, 2011; Isaiah, Vongpaisal, King, & Hartley, 2014; Keating, Dahmen, & King, 2013; Nodal, Bajo, & King, 2012; Nodal et al., 2010).

Using a combination of operant- and reflex-based paradigms, behavioral gap-detection thresholds and response parameters have been well-described in mice (Ison, Allen, Rivoli, & Moore, 2005; Ison, Castro, Allen, Virag, & Walton, 2002; Radziwon et al., 2009), rats (Ison, O’Connor, Bowen, & Bocirnea, 1991; Leitner et al., 1993; Lobarinas, Hayes, & Allman, 2013; Rybalko & Syka, 2005; Syka, Rybalko, Mazelová, & Druga, 2002; Turner et al., 2006), gerbils (Hamann, Gleich, Klump, Kittel, & Strutz, 2004; Wagner, Klump, & Hamann, 2003), hamsters (Chen et al., 2013; Salloum, Yurosko, Santiago, Sandridge, & Kaltenbach, 2014), guinea pigs (Dehmel, Eisinger, & Shore, 2012; Koehler & Shore, 2013), and chinchillas (Giraudi, Salvi, Henderson, & Hamernik, 1980; Salvi & Arehole, 1985) and have been characterized in a variety of nonmammalian species, including reptiles, amphibians, and birds (e.g., Dooling, Lohr, & Dent, 2000; Dooling, Zoloth, & Baylis, 1978; Höbel, 2014; Klump & Maier, 1989; Okanoya & Dooling, 1990). Gap-detection psychophysical experiments have also been the focus of extensive lines of enquiry in normal-hearing human subjects (Allen, Virag, & Ison, 2002; Buunen & van Valkenburg, 1979; Formby, Gerber, Sherlock, & Magder, 1998; Horwitz, Ahlstrom, & Dubno, 2011; Oxenham, 2000; Penner, 1977; Phillips & Hall, 2000; Phillips, Taylor, Hall, Carr, & Mossop, 1997; Plomp, 1964; Samelli & Schochat, 2008; Shailer & Moore, 1983, 1985; Snell & Hu, 1999; Snell, Ison, & Frisina, 1994). By contrast, little is known about the perceptual correlates of gap-in-sound processing in ferrets, apart from a single study comprising two comprehensively tested animals that displayed heterogeneous performance (Kelly, Rooney, & Phillips, 1996).

In the present study, we sought to establish gap-detection behavioral parameters in the ferret using an operant conditioning paradigm. In particular, we aimed to measure detection thresholds and related behavioral parameters (including approach-to-target and head-orienting dynamics) in response to spectrally varied stimuli presented in the free field. To verify the use of the ferret as a suitable model of human auditory processing, we also tested a cohort of human listeners on a similar psychophysical task. Our results show that ferrets perform well in operantly trained gap detection, with detection thresholds that are comparable to those previously described in this species, as well as other smaller mammals trained on similar tasks. Moreover, thresholds and performance, as well as unconditioned head-orienting behavior, were found to be affected by the stimulus frequency content. Comparable observations were made in our human subjects, establishing a means by which to compare temporal processing in the two species.

## Method

All experiments were carried out in sound-attenuated chambers and were approved by local ethical review committees and, in the case of ferret psychophysical experiments, were performed following ethical review by the Committee on Animal Care and Ethical Review of the University of Oxford and under license from the UK Home Office in accordance with the Animal (Scientific Procedures) Act (1986, amended in 2012).

### Animals and Welfare

Nine adult pigmented female ferrets (*Mustela putorius furo*; age range: 5–24 months) were used in these experiments. Ferrets were housed in small groups in standard laboratory cages equipped with behavioral enrichment. During periods of behavioral testing, animals were motivated to perform the gap-detection task by water access regulation, with each period of testing lasting a maximum of 5 days, with two testing sessions per day. In these testing periods, ad libitum access to dry pelleted food was provided, whereas daily water requirements were obtained through correct performance of the task alongside supplementation (in the form of a pellet–water mash) following completion of each day’s testing. Animal body weights were monitored routinely and compared with that individual’s baseline measurements, which were obtained prior to starting each testing period. Typically, body weights fell by <5% during each testing period. Between each testing period, animals were allowed ≥2 days during which ad libitum access to water was provided. Otoscopic examinations and tympanometry were performed periodically to assess the status of the outer and middle ear of each animal. Measurement of auditory brainstem responses to verify central auditory function was conducted for each animal, as in previous studies (Moore, 1993; Morey & Carlile, 1990), with ferrets showing responses in line with normative veterinary data (Piazza, Huynh, & Cauzinille, 2014; data not shown).

### Testing Apparatus and Stimuli

**Figure 1 fig1:**
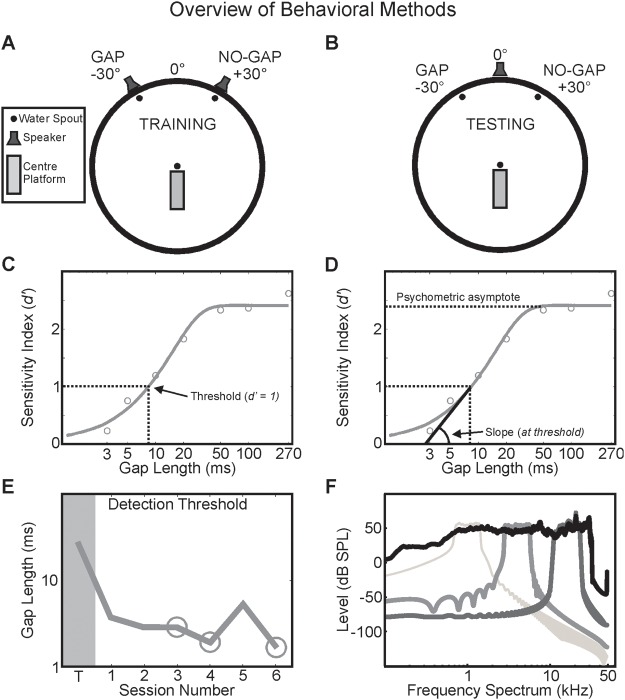
Overview of behavioral methods. Ferrets were trained using a positive operant conditioning paradigm to report the perceived presence of a silent gap in a noise stimulus by nose-poking at one of two reward spouts at the periphery of a circular testing chamber (A). Trials were initiated by standing on a center platform, facing toward 0°, and nose-poking a central reward spout. During the training phase, “gap” stimuli were presented from a loudspeaker above the spout at −30°, whereas “no-gap”/continuous noise stimuli were presented from a loudspeaker located at +30°. During the testing phase, once the stimulus types (gap vs. no gap) were reliably paired with rewards at specific locations, all stimuli were presented from a loudspeaker at 0°, while responses were registered at water spouts positioned at ±30° (B). Example performance of one ferret during a session of the gap-detection task for the broadband noise stimulus (C). The percentage of correct responses for each gap length was used to construct psychometric functions, allowing estimation of the gap-detection threshold. Psychometric functions were transformed according to signal detection theory (Macmillan & Creelman, 2005) into sensitivity (*d*′), using the fitted psychometric functions, including empirical false alarm rates, on a session-by-session basis. Threshold was defined as the estimated gap length corresponding to a sensitivity of *d*′ = 1. The slope was computed as the gradient of the psychometric curve at threshold sensitivity (D). The asymptote of the curve was used to compute the maximum sensitivity, while the lapse rate was obtained experimentally. Example of gap-detection threshold progression as a function of session number for one animal (E). Data collected during training were not included in the analysis (T, dark gray block). Performance was stable over time, with the best three sessions extracted over the entire testing period (circles). Power spectra of the noise stimuli used in this experiment, comprising octave–bandpass narrowband noise (NBN) centered at 1 kHz (light gray), 4 kHz (midgray), or 16 kHz (dark gray) or flattened broadband noise (BBN) with a 30-kHz low-pass corner (black; F). Color scheme retained throughout figures.

Gap-detection behavioral testing was carried out in a circular arena (see Figure 1) with a radius of 70 cm with a solid plastic floor and enclosed by a cylindrical mesh barrier, which was located inside a double-walled testing chamber lined with 50-mm acoustic foam (MelaTech, Hodgson & Hodgson Ltd., Melton Mowbray, UK). Animals were monitored from an external room via a closed-circuit monitor. At the center of the testing arena, a rectangular raised platform was present with a steel spout and an optical proximity sensor located at the rostral end (defined as 0°). In order to initiate a trial, the animal was required to stand on the platform and nose-poke the central spout. Spout contact and the required animal orientation were ensured by the coactivation of the center spout sensor and an infrared beam sensor located at the rear corners of the platform. Upon trial initiation, an acoustic stimulus was presented at the chamber’s periphery from either one of two loudspeakers (Figure 1A) or a single loudspeaker (Figure 1B; FRS 8, Visaton, Crewe, UK), which were occluded from sight by muslin cloth. Animal responses were registered at two lateralized water spouts (situated at the arena periphery at a bearing of ±30° relative to center), with correct responses rewarded by a fixed amount of water. Following incorrect responses, the animal was required to reinitiate the trial (“correction trials”), with only the first response included in the data analysis. Stimulus presentation, response registration, and water rewards were controlled using TDT System III hardware (Tucker-Davis Technologies, Alachua, Florida), with all experimental contingencies controlled using custom-written scripts implemented in MATLAB (MathWorks, Natick, Massachusetts).[Fig-anchor fig1]

Stimuli were generated de novo on each trial using TDT System III hardware. For each testing session, stimuli (all 2,080 ms in duration) were either broadband noise bursts (low pass filtered up to 30 kHz; BBN) or one of three narrowband noise burst types (bandpass filtered with an octave window logarithmically centered at 1, 4, or 16 kHz; NBN) and stopband rolloff of at least −52 dB/octave. All stimuli were filtered using the inverse transfer function of the speaker to ensure a flat response across the stimulus frequency spectrum, with the overall level averaging 76 ± 5 decibels sound pressure level (dB SPL) from trial to trial, irrespective of stimulus type (Figure 1F).

### Behavioral Training

During initial training, animals were familiarized with the testing arena and taught to approach the central spout with the correct orientation (facing 0°), for which they received a water reward of multiple drops of water; over time (and prior to beginning behavioral testing), this center reward was reduced to one drop every 20 trials (pseudorandomized with a reward probability of 0.05). Once trained, center spout approach was followed (at a delay slowly increased up to 500–1,000 ms of maintained spout contact) by broadband stimulus presentation at one of two loudspeakers, each situated behind the two water spouts at a ±30° bearing (Figure 1A). Stimuli presented from the speaker at +30° were continuous noise bursts (“no gap”); stimuli presented from the loudspeaker at −30° were noise bursts into which four silent gaps had been interleaved (gap duration of 270 ms; 2-ms cosine ramp; “gap”). Gaps were interleaved so that their centers were evenly spaced throughout the noise stimulus, which did not change in overall duration between the gap and no-gap conditions, thereby producing five distinct noise bursts, each of duration [2,080 − (4 × gap length)]/5 ms. Stimuli were pseudorandomly presented in a 1:1 ratio of gap:no gap.

Once an animal’s task performance was consistently >90% correct, presentation of each stimulus type was switched to a single loudspeaker at 0° azimuth (Figure 1B), with the duration of the silent gaps in gap stimuli comprising 50, 100, or 270 ms. Correct responses for gap and no-gap stimuli continued to be registered at the water spouts situated at −30° and +30°, respectively, and rewarded with three drops of water (this remained consistent throughout behavioral testing).

### Behavioral Testing

Once task performance was consistently >90% correct, the tested set of gap lengths was expanded to include 3, 5, 10, 20, 50, 100, and 270 ms (by association, the corresponding noise bursts for each gap length were 413.6, 412, 410, 400, 376, 336, and 200 ms, respectively). The gap length on each trial was selected from this distribution and pseudorandomly varied on a trial-by-trial basis. The gap:no-gap stimulus presentation ratio remained at 1:1, with gap/no-gap stimulus type also determined on a pseudorandom trial-by-trial basis. Daily testing was conducted until at least 1,000 trials were cumulatively collected for broadband stimuli. Following this, testing continued with each session’s stimulus frequency content randomly chosen between the three narrowband stimulus types, until >1,000 trials had been collected for each one. On each trial, the animal’s approach-to-target response time was registered, equating to the time between stimulus onset and peripheral spout licking. Trials in which a center spout reward was provided were not included in the final analysis.

### Head-Orienting Responses

Due to the lateralized nature of the initial two-speaker training protocol and the left–right response registration method, we measured the change in each animal’s head orientation to ensure that a latent sound localization behavior was not biasing the results. These measurements were obtained as described previously (Nodal et al., 2008). Briefly, an infrared-sensitive camera was positioned above the central spout and used to track the coordinates of a self-adhesive reflective strip, attached to the midline of the animal’s head, at a rate of 60 frames/s. From these data, the angular extent of the animal’s head turning was calculated with respect to its initial head bearing. A head-turning response was defined as a period of more than three consecutive frames over which the animal’s head moved in the same rotational direction, while initial head-turning response time was defined as the time of the first frame of this response. The final head bearing was measured as the mean of the last three frames of the initial continuous head movement or, if a head movement was not recorded, as the mean of the final three frames recorded prior to the animal moving from the platform. Trials were excluded from analysis if the initial head bearing was more than 2 standard deviations from the population mean initial head bearing.

### Human Psychophysics

We tested six human adults (four females, two males; mean age of 29 years) with audiograms performed prior to testing; subjects’ thresholds were confirmed as normal up to and including 16 kHz, with no impairments in any of our subjects tested. Gap-in-noise detection performance was evaluated using stimuli of the same spectral type as described for the ferret experiments: broadband noise (low pass 30 kHz) and 1-octave narrowbands centered at 1, 4, and 16 kHz at an intensity of 76 ± 5 dB SPL (Figure 1F). Stimuli differed from those used for testing ferret gap detection in two ways: Stimulus noise duration was 400 ms, and only a single gap was interleaved within the acoustic stimulus. Gap lengths used were 2, 5, 10, and 20 ms during an initial training session and 1, 2, 3, 4, and 10 ms during testing. Subjects were seated in a double-walled sound-attenuating chamber, with stimuli presented from a single loudspeaker (Audax TW025M0) located at 0° at eye level.

Responses were recorded via a graphical user interface either using a keyboard by pressing the keys “1” and “2” or mouse clicks on labeled onscreen buttons for a gap and no-gap response, respectively. No feedback was provided during testing. In the case of incorrect responses, correction trials were not provided. For each testing session (of which three were conducted for each stimulus type), data were collected in independent blocks of 100 trials for the different stimulus types, with stimulus frequency content pseudorandomized from block to block. Within each block of trials, gap/no-gap content and gap length were pseudorandomized as for ferret behavioral testing. All experimental contingencies were controlled by custom-written scripts in MATLAB and implemented in TDT System III hardware.

### Data Analyses

All data were analyzed using MATLAB. For gap-detection responses, percent-gap functions (equivalent to percent correct functions, except with no-gap percent incorrect values included to anchor the function at gap length = 0 ms; i.e., no gap) were calculated for each session. Sessions were excluded from analysis if fewer than five different gap lengths were tested <5 times within that session. Psychometric functions for percent-gap values were fitted using the open-source package psignifit (http://psignifit.sourceforge.net; Figure 1C). Fit parameters were specified according to a right-weighted gumbel sigmoid fitted over a linear transform of the stimulus intensity values (“mw0.1” core). Goodness of fit was determined for each session’s psychometric function by bootstrapping the fit deviance to generate a reference distribution of 1,000 sample sets. Fits exceeding the 95th percentile of the bootstrapped deviance population were discarded from subsequent analysis, according to criteria recommended by Fründ, Haenel, and Wichmann (2011). For each psychometric function, percent-gap values were transformed into *d*′ values according to signal detection theory (SDT) (Macmillan & Creelman, 2005), a theoretical framework that has been previously validated in an operantly conditioned yes/no-type paradigm in ferrets (Alves-Pinto et al., 2012). *d*′ values were obtained using the transform d’=z(Hit)−z(FA), where *Hit* and *FA* are the hit and false alarm rates; that is, the proportion of approach-to-target responses made correctly for gap stimuli and incorrectly for no-gap stimuli, respectively. From the transformed psychometric functions, we were able to extract various measures of performance that have been described previously (Buran et al., 2014). Performance threshold was defined as the fitted gap duration at which *d*′ = 1, which approximates a percent-gap rate of approximately 60%, given the mean population false alarm rate (FA rate *M* = 0.23) recorded across all stimulus types. Psychometric function slope was calculated at threshold to determine the rate at which an animal’s session performance approached asymptote performance. In addition, we calculated two measures of optimal performance: the lapse rate, which is the observed proportion of incorrect responses obtained at the longest gap duration, and maximum sensitivity, which is the *d*′ value at each function’s asymptote (Figure 1D).

As previously described (e.g., Lacouture & Cousineau, 2008; Leach, Nodal, Cordery, King, & Bajo, 2013), the distributions of approach-to-target and head-orienting response times in operantly conditioned tasks of this kind are nonnormal and are best approximated using an ex-Gaussian function, each of which was fitted using the DISTRIB toolbox (V2.3; http://darwin.psy.ulaval.ca/~yves/distrib.html). Statistical tests were performed on the fit parameters of each distribution: μ and σ (equivalent to the mean and standard deviation of the Gaussian component of the distribution) and τ (equivalent to the time constant of the distribution’s exponential component).

### Statistical Analysis

Statistical tests were conducted by generating a generalized multilevel mixed-effects model of raw data, according to a Bernoulli distribution, in R (www.r-project.org). Tests for the significance of derived behavioral measures were performed in MATLAB using multilevel analyses of variance (ANOVAs), with subject identity and testing session included as nested random factors. Post hoc tests were Tukey-corrected for multiple comparisons. Data are reported as mean ± standard error (*SE*).

In order to assess the significance of correlations between threshold and slope, a bootstrap procedure was employed. Here, the between-groups correlation statistic (Spearman’s ρ) was first calculated (for threshold vs. slope across stimuli and subject). Pairs of data points were then resampled with a replacement from each randomly shuffled set, and the correlation was computed. This procedure was repeated 10,000 times to generate a reference distribution, from which the two-sided *p* value of the actual statistic was computed.

## Results

### Behavioral Performance

Gap-detection sensitivity was measured by the gap length at which the psychometric function for each session reached the threshold, defined here as *d*′ = 1. This sensitivity level was chosen to simplify comparison with other nonhuman behavioral paradigms (Macmillan & Creelman, 2005). The mean false alarm (FA) rate across all tested animals for the broadband stimulus was 0.23; therefore, to achieve *d*′ = 1, an equivalent hit rate of 0.61 was required. These parameters correspond to an equivalent corrected performance ([Hit rate − FA rate]/[1 − FA rate]) of 0.5, which has previously been used for threshold estimation (Kelly et al., 1996). Notably, detection thresholds remained fairly constant from session to session, ruling out any effect of learning beyond the initial training phase (Figure 1E).

**Figure 2 fig2:**
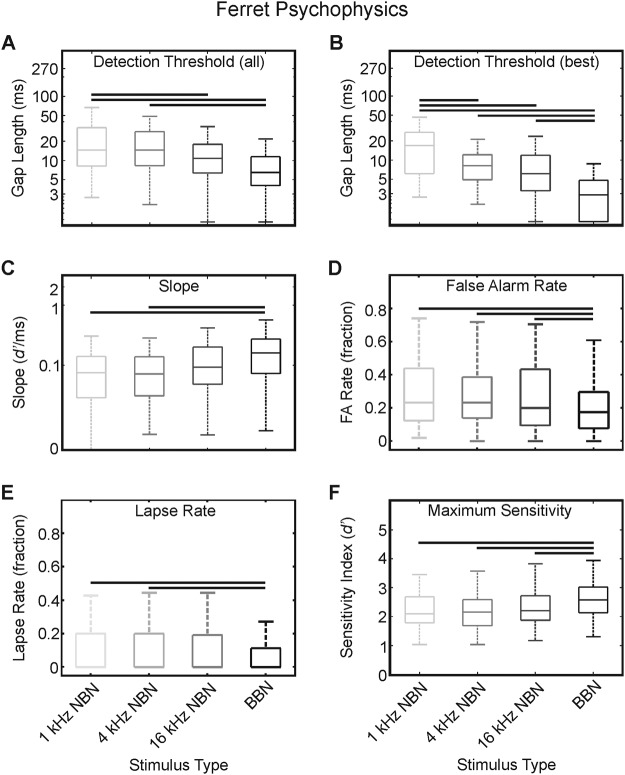
Ferret behavioral gap-detection results. Gap-detection threshold measured as gap length necessary to achieve a sensitivity of *d*′ = 1, measured across stimulus types (A). In all figures, data plotted comprise all sessions across all subjects, where box plots indicate medians and interquartile ranges in each condition, with whiskers illustrating maxima and minima. Box outline colors correspond to stimulus frequency: light gray = 1-kHz NBN; midgray = 4-kHz NBN; dark gray = 16-kHz NBN; black = BBN. Horizontal bars indicate significant pairwise post hoc difference at *p* < .05, corrected for multiple comparisons. Gap-detection thresholds calculated from each animal’s three best sessions (B). Psychometric function slope at threshold (C). False alarm rates, equivalent to proportion of incorrect trials for no-gap stimuli (D). Lapse rates, equivalent to proportion of incorrect trials for stimuli with gap length = 270 ms (E). Maximum sensitivity, measured as *d*′ at the asymptote of each session’s psychometric function (F).

Ferrets displayed significant interindividual differences in performance irrespective of testing conditions, *F*_(8, 390)_ = 15.82; *p* < .001. Nevertheless, when testing for stimulus spectrum-dependent differences in the gap-detection threshold and controlling for random effects of animal identity on behavioral performance, there emerged a significant main effect of stimulus type on gap length at *d*′ = 1, *F*_(3, 390)_ = 13.21; *p* < .001; Figure 2A. Tukey-corrected post hoc comparisons of stimulus type indicated that animals were markedly more sensitive to gaps presented in broadband (threshold [ms] = 11.93 ± 1.26) or high-frequency (16 kHz) narrowband noise (threshold [ms] = 16.40 ± 1.73), relative to narrowband noise centered at 1 kHz (threshold [ms] = 24.56 ± 2.00) or 4 kHz (threshold [ms] = 21.83 ± 1.66). To ensure that our session-by-session analysis protocol was not yielding abnormal results, we reanalyzed our data as sets of all trials compiled across those testing sessions included in the above analysis. In this case, a significant main effect of stimulus type was still observed, *F*_(3, 21)_ = 3.63; *p* = .02, with frequency group thresholds (ms, mean ± *SE*) of 1-kHz NBN = 29.3 ± 10.4; 4-kHz NBN = 20.0 ± 3.4; 16-kHz NBN = 15.7 ± 3.1; and BBN = 10.4 ± 2.7. Notably, these estimates are within the error limits provided in our session-by-session analysis.[Fig-anchor fig2]

We further explored whether the differential sensitivity to gap length with stimulus type could be accounted for by individual variations in performance, as suggested by the interaction between ferret identity and stimulus type, *F*_(8, 24)_ = 10.07; *p* < .001. This interaction suggests that our animals displayed heterogeneous performance akin to that observed by Kelly et al. (1996). To do this, we restricted our analysis to include only those data from each animal’s three best sessions (Figure 1E), which is likely to reduce the within-subject variability, as indicated previously (Buran et al., 2014). From this analysis, a significant main effect of stimulus remained, *F*_(3, 72)_ = 4.09; *p* = .018 (Figure 2B), whereas a main effect of ferret was not observed, *F*_(8, 72)_ = 0.97; *p* = .48. Post hoc comparisons revealed significant differences in performance between all stimuli (*p* < .05, Tukey corrected), except between 4 and 16 kHz (threshold [ms], mean ± *SE*: 1-kHz NBN = 18.19 ± 2.56; 4-kHz NBN = 11.03 ± 1.98; 16-kHz NBN = 8.31 ± 1.17; BBN = 4.22 ± 0.89).

This result suggests that each animal was operating within a similar behavioral range in their respective best sessions, though it gives no insight into the variability in subject performance. Therefore, to further evaluate the degree to which ferrets differed in their session-to-session performance, we calculated the standard deviation of thresholds collected on each session for each subject for each stimulus type. This provides an estimate of the variability from session to session of ferret performance. For each stimulus, these data were found to be (mean ± *SE*) 1-kHz NBN, 14.6 ± 4.6; 4-kHz NBN, 13.3 ± 3.4; 16-kHz NBN, 10.8 ± 3.1; and BBN, 10.1 ± 4.2. None of these values was different statistically from any other (Kruskal–Wallis, χ^2^ = 3.24, *df* = 3, 35, *p* = .36). In light of the significant Ferret × Stimulus interaction, this indicates that all animals are performing within a similar range across stimulus types, though individual animals perform better or worse in general when accounting for all analyzed sessions.

To investigate whether stimulus type affected the degree to which ferrets were sensitive to changes in gap length, we measured the slopes of the psychometric functions by differentiating the curve at the computed threshold level for that session (Figure 1D). An inverse relationship was found between gap length and slope at threshold, with a stimulus-dependent main effect on psychometric function steepness observed, *F*_(3, 390)_ = 4.59; *p* = .004 (Figure 2C). When investigated using pairwise comparisons of stimulus type, significantly steeper psychometric functions were recorded for broadband stimuli than for 1- and 4-kHz narrowband noise (slope [*d*′/ms], mean ± *SE*: 1-kHz NBN = 0.10 ± 0.04; 4-kHz NBN = 0.13 ± 0.03; 16-kHz NBN = 0.14 ± 0.04; BBN = 0.25 ± 0.03). These data demonstrate that for naïve ferrets, smaller differences in gap length could be better discriminated when stimuli comprised flattened broadband noise compared with narrowband stimuli that were centered at the lower end of the ferret’s audiometric hearing range.

In order to quantify the relationship between threshold and slope, we calculated the correlation for threshold versus slope across all data points, irrespective of subject or stimulus type. We found a strong negative correlation between gap-detection threshold and slope (Spearman’s rank correlation, ρ = −0.86, *p* < .0001). This would indicate that where thresholds are low, they are accompanied by greater sensitivity to small differences in stimulus increment.

In addition to gap length sensitivity at threshold, we computed three additional measures of the ferret’s operant performance. First, we calculated the false alarm rate (the proportion of no-gap trials for which animals reported the presence of a gap in noise), which in SDT depends on the subject’s internal decision criterion and varies with the set of stimulus conditions (Figure 2D). A significant main effect of stimulus type emerged when a mixed-effects ANOVA was conducted, *F*_(3, 390)_ = 6.52; *p* < .001; this result was mainly explained by the large pairwise differences between broadband versus narrowband stimuli, with the former yielding significantly lower FA rates compared with all narrowband sounds (FA rate [fraction], mean ± *SE*: 1-kHz NBN = 0.28 ± 0.02; 4-kHz NBN = 0.29 ± 0.01; 16-kHz NBN = 0.28 ± 0.01; BBN = 0.23 ± 0.01).

To investigate aspects that might be influencing the ferret’s decision-making process, two parameters were calculated to describe the upper end of the psychometric function: the lapse rate (the proportion of incorrect responses registered for a gap width of 270 ms) and the behavioral sensitivity at asymptote (Figure 1D). Each measure is related to the optimal performance that might be expected from an individual animal for each stimulus type—for example, high lapse rates for easy stimuli could be indicative of a general lack of attention. For both parameters, a main effect of stimulus type was highly significant in accounting for between-condition differences—lapse rate: *F*_(3, 390)_ = 9.35; *p* < .001; maximum sensitivity: *F*_(3, 390)_ = 18.78; *p* < .001 (Figures 2E and 2F)—suggesting that performance was indeed influenced by one or more of these factors in a stimulus-dependent fashion.

For the lapse rate data, significant differences between broadband and narrowband stimuli emerged, with lapse rates in response to BBN much lower compared with 1- and 4-kHz NBN conditions (lapse rate [fraction], mean ± *SE*: 1-kHz NBN = 0.13 ± 0.02; 4-kHz NBN = 0.12 ± 0.01; 16-kHz NBN = 0.09 ± 0.01; BBN = 0.05 ± 0.01; Figure 2E). With respect to sensitivity at the asymptote of the psychometric curve (Figure 1D), post hoc tests indicated that animals were more sensitive to broadband noise than to any of the narrowband stimuli (maximum sensitivity [*d*′], mean ± *SE*: 1-kHz NBN = 2.07 ± 0.08; 4-kHz NBN = 2.12 ± 0.06; 16-kHz NBN = 2.28 ± 0.07; BBN = 2.61 ± 0.05; Figure 2F). Each of these measures represents a combination of the lower hit rates and higher FA rates obtained with narrowband versus broadband stimuli, particularly those centered at lower sound frequencies. The similarity in the observed effects of stimulus on both lapse rate and asymptote sensitivity is captured in the moderate but significant correlation between them (Spearman’s rank correlation, ρ = −0.35, *p* < .0001).

We can thus conclude that the ferret’s gap-in-noise detection performance is significantly affected by the type of stimulus used as a noise carrier in the operant task; in particular, ferrets were found to display significantly better behavioral performance and sensitivity to silent gaps interleaved within broadband noise relative to narrowband noise stimuli, with the greatest differences seen for low- and midfrequency-centered NBN.

### Behavioral Response Times

In previous reports of ferret operant behavior, response times—computed as the time between stimulus onset and response registration—have been measured and correlated with task difficulty as well as with changes in auditory cortical physiology (Bajo, Nodal, Moore, & King, 2010; Leach et al., 2013; Nodal et al., 2008, 2010). Indeed, response times in general are thought to provide a measure of the effects of attention in behavioral paradigms (Buck, 1966; Buran et al., 2014; Luce & Green, 1972; Salthouse & Hedden, 2002; Saltzman & Garner, 1948).

**Figure 3 fig3:**
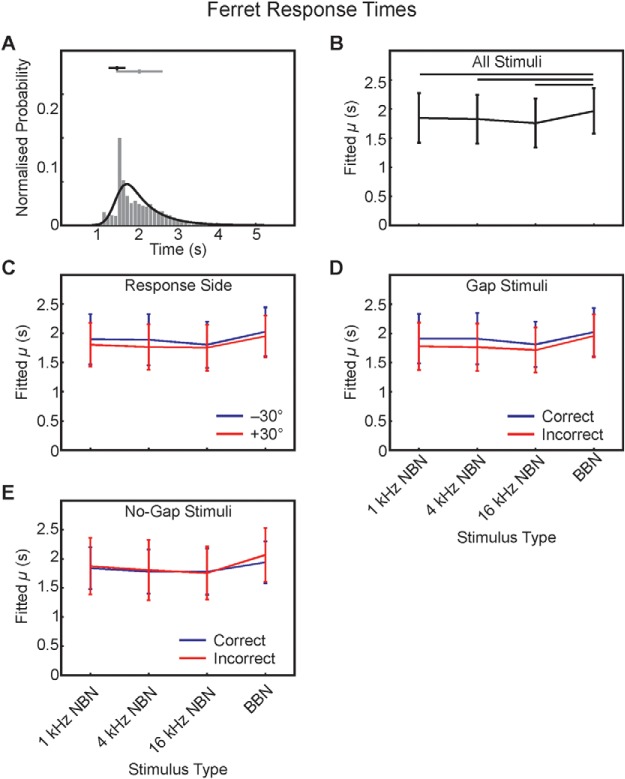
Ferret response time data. Example normalized histogram of response times for all trials performed by one animal in response to 1-kHz NBN (A). Values were binned at 100 ms and indicate the time between stimulus onset and a nose-poke response being made at the periphery (at either reward spout). An ex-Gaussian distribution (black) was used to model these data in MATLAB using the DISTRIB toolbox available from http://darwin.psy.ulaval.ca/~yves/distrib.html. Markers with horizontal bars indicate mean ± standard deviation (gray) of the data and μ ± σ (black) values of the fitted distribution. μ as a function of stimulus type for all trials collected from all animals; line plots indicate mean ± *SE* across all data in each case (B). Horizontal bars indicate significant pairwise post hoc difference at *p* < .05, corrected for multiple comparisons. μ values subdivided according to whether the approach-to-target response made to the left/−30° reward spout (blue) or the right/+30° reward spout (red; C). Between-groups differences significant at *p* = .013. μ values from gap stimuli trials, subdivided into correct (blue) and incorrect (red) responses (D). Between-groups differences significant at *p* = .004. μ values from no-gap stimuli trials, subdivided into correct (blue) and incorrect (red) responses (E). Between-groups differences not significant, *p* = .30.

As in previous studies (Lacouture & Cousineau, 2008; Leach et al., 2013), we found that the response time distributions were highly nonnormal and were well fitted using an ex-Gaussian function (Figure 3A), and statistics were performed on the mean of the Gaussian component of the fitted curve (μ), representing the shorter-latency component of the response time distribution. Evaluating these data by mixed-effects modeling, we found a significant main effect of stimulus on response times, *F*_(3, 15)_ = 15.24; *p* < .001. Pairwise post hoc testing indicated this effect to be due to significantly longer μ values during broadband testing, with no differences observed between narrowband conditions (μ [s], mean ± *SE*: 1-kHz NBN = 1.85 ± 0.43; 4-kHz NBN = 1.83 ± 0.42; 16-kHz NBN = 1.76 ± 0.42; BBN = 1.97 ± 0.39; Figure 3B).[Fig-anchor fig3]

We also examined the effects of the location of the response spouts and the differences in correct versus incorrect response locations for gap versus no-gap stimuli on response times. When we investigated the effect of the direction in which the animals made their response (±30°), irrespective of stimulus spectrum, and collapsing across gap/no-gap stimulus type, we found a significant main effect on response time μ, *F*_(1, 38)_ = 6.78; *p* = .013, with shorter μ values for the spout at +30° for all conditions (μ [s], mean ± *SE*: left = 1.90 ± 0.40; right = 1.81 ± 0.36; Figure 3C). Given that rightward responses correspond to incorrect responses in the gap condition and to correct responses in the no-gap condition, it is perhaps unsurprising that a similar main effect was found when factoring in stimulus type (gap or no gap) and response outcome (correct vs. incorrect), such that incorrect gap responses were significantly faster than correct gap stimulus responses, *F*_(1, 38)_ = 9.60; *p* = .004; μ (s), mean ± *SE*: correct = 1.91 ± 0.40; incorrect = 1.80 ± 0.38 (Figure 3D). Interestingly, such effects were not seen for μ values in response to no-gap stimuli, *F*_(1, 38)_ = 1.12; *p* = .30; μ (s), mean ± *SE*: correct = 1.84 ± 0.36; incorrect = 1.88 ± 0.46 (Figure 3E). These data suggest that the ferrets exhibited a stereotyped behavior directed toward the rightward no-gap response spout, with correct gap responses made as additional information was obtained throughout the duration of the stimulus; by comparison, incorrect no-gap trials are likely to have been initiated from trial onset rather than in the period after the ferret had begun to make its response.

### Head-Orienting Behavior

To rule out any possibility that the animals’ responses to each reward spout might reflect the lateralized nature of the initial training protocol, it was necessary to evaluate unconditioned head-orienting responses, since previous studies of ferret operant behavior have demonstrated a clear relationship between approach-to-target sound localization behavior accuracy and head-orienting response accuracy (Leach et al., 2013; Nodal et al., 2008). In the present study, we sought to determine the extent to which this unconditioned behavior in response to stimulus location might have been influenced by the use of stimulus lateralization to bind gap/no-gap identity to specific response spouts during the training phase (Figure 1A).

**Figure 4 fig4:**
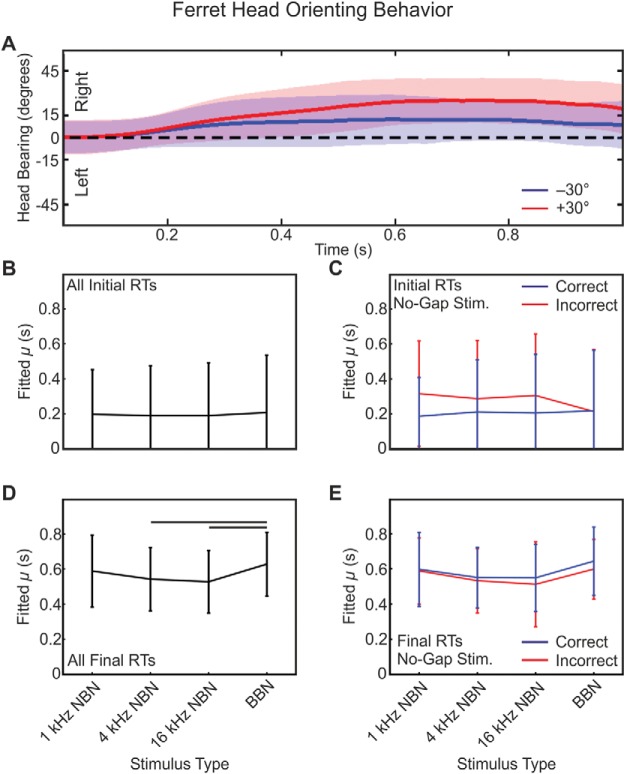
Sound-evoked head-orienting responses. Horizontal head angle trajectory from one animal as a function of time over the course of BBN stimulus presentation for all gap lengths for responses made to the left (blue) or right (red) reward spout (A). Traces are mean (thick line) ± *SE*. Ordinate values are positive for rightward bearing. Head-orienting latency data, like response time data, were well fitted using an ex-Gaussian distribution, with statistics performed on fitted μ. μ (in seconds) for initial head-orienting latency as a function of stimulus type (B). Line plots indicate reaction time (RT) mean ± *SE* in each case. μ for initial head-orienting latency for no-gap trials, subdivided into correct (blue) and incorrect (red) responses (C). Between-groups differences significant at *p* = .042. μ for termination of head-orienting response (D). Horizontal bars indicate significant pairwise post hoc difference at *p* < .05, corrected for multiple comparisons. μ for termination of head-orienting response for no-gap trials, subdivided into correct (blue) and incorrect (red) responses (E).

Recordings of head-orienting behavior yielded a stereotyped angular displacement over time toward the right regardless of left–right response location—final head bearing: *F*_(3, 10,689)_ = 0.03; *p* = .993 (example shown in Figure 4A). This indicates that the lateralized operant response training phase, in which a pair of loudspeakers was positioned at ±30°, did not have an effect on the orienting responses when a single loudspeaker was used. Consequently, final head bearing cannot be used as a predictor of which reward location the animals approached. However, the observed bias toward the right, which may simply indicate a preference for leaving the platform from this side of the center spout, could partially explain why approach-to-target responses to the right were quicker, since the head-orienting response presumably comprises the initial component of the approach response.[Fig-anchor fig4]

Analysis of the response times from the unconditioned head-orienting responses to stimuli located at 0° azimuth can provide insight into the decision–detection process. As was the case for the approach-to-target response times described above, head-orienting response latency data were well fitted using ex-Gaussian distributions. We measured the latencies both for the initial head movement (initial) and at the point when the head movement terminated or the animal left the central platform (final). Initial head-bearing latencies were not significantly affected by stimulus type, *F*_(3, 15)_ = 0.41; *p* = .75; μ (ms), mean ± *SE*: 1-kHz NBN = 196.79 ± 255.16; 4-kHz NBN = 189.20 ± 285.96; 16-kHz NBN = 189.13 ± 302.48; BBN = 206.79 ± 328.91 (Figure 4B). However, a marginally significant effect on μ was found for no-gap stimuli when these responses were subdivided according to trial correct–incorrect outcome, *F*_(1, 38)_ = 4.41; *p* = .042 (Figure 4C). In contrast, final-response latency-fitted μ was significantly greater for broadband stimuli than for 4- or 16-kHz NBN, *F*_(3, 15)_ = 6.50; *p* = .005; fitted μ (ms), mean ± *SE*: 1-kHz NBN = 588.66 ± 205.65; 4-kHz NBN = 542.44 ± 180.00; 16-kHz NBN = 527.30 ± 178.36; BBN = 628.28 ± 181.45 (Figure 4D), but no differences were found for no-gap stimuli between correct and incorrect responses, *F*_(1, 38)_ = 3.76; *p* = .060 (Figure 4E).

### Human Psychophysics

**Figure 5 fig5:**
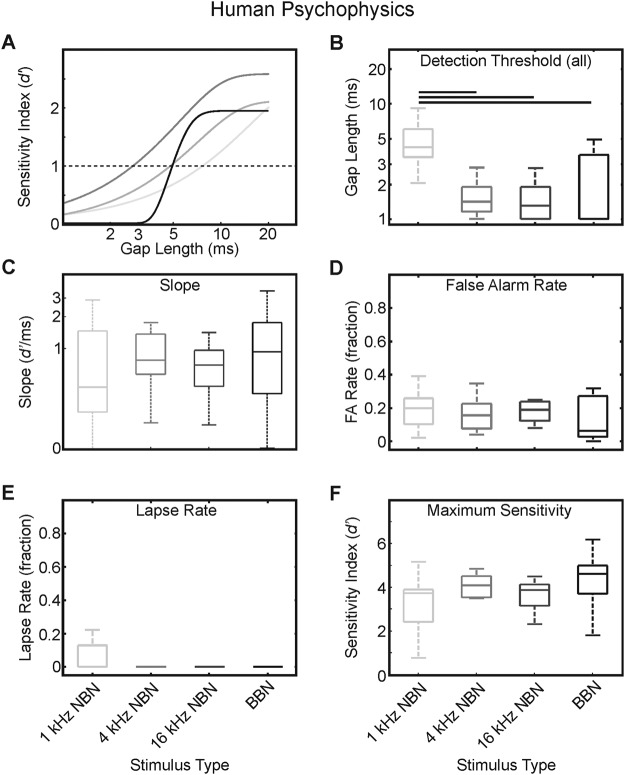
Human cohort psychometric gap-detection behavioral results. Example psychometric functions for one subject plotted as sensitivity (*d*′) for each stimulus type (A). Trace colors correspond to stimulus frequency: light gray = 1-kHz NBN; midgray = 4-kHz NBN; dark gray = 16-kHz NBN; black = BBN. For visualization of parameters extracted from psychometric functions, refer to Figures 1C and 1D. Gap-detection thresholds (B). Box plots indicate medians and interquartile ranges in each case, with whiskers illustrating maxima and minima. Horizontal bars indicate significant pairwise post hoc difference at *p* < .05, corrected for multiple comparisons. Psychometric function slopes at threshold (C). False alarm rates (D). Lapse rates (E). Note that no lapses were made by any subject during any session for the 4-kHz NBN, 16-kHz NBN, and BBN stimulus conditions. Maximum sensitivity at psychometric function asymptote (F).

To evaluate the utility of the ferret as a reliable and accurate model of human auditory processing, we tested human subjects on a gap-detection task using identical spectral filters to generate sound stimuli. Although response times were not measured, correct–incorrect response type data were exposed to identical analyses to those conducted for the animal behavioral results to facilitate comparison with respect to psychometric function features (Figure 5A). Across the cohort tested, thresholds for gap-in-noise resolution were mostly <10 ms (gap length at *d*′ = 1 [ms], mean ± *SE*: 1-kHz NBN = 5.21 ± 2.78; 4-kHz NBN = 1.76 ± 1.00; 16-kHz NBN = 1.72 ± 1.13; BBN = 2.15 ± 1.58). As was observed in our ferret cohort, human subjects displayed a significant main effect of stimulus type on detection threshold at *d*′ = 1, *F*_(3, 45)_ = 15.79; *p* < .001 (Figure 5B). This was accounted for by a significant difference between 1-kHz narrowband detection thresholds and those recorded for all other stimulus bandwidths. Although there was no main effect of slope (Figure 5C), we did find a moderate but significant correlation between detection threshold and slope for our human subjects (Spearman’s rank correlation, ρ = −0.45, *p* = .0007), in line with the relationship between threshold and incremental sensitivity seen in ferrets. For each FA rate (Figure 5D) and asymptote sensitivity (Figure 5F), we did not find a significant main effect of stimulus type. It is notable that the lapse rates for all stimuli were exceptionally low (Figure 5E), with no lapses made at all for the broadband 4- and 16-kHz conditions, thus indicating the highly attentive state of our subjects.[Fig-anchor fig5]

**Figure 6 fig6:**
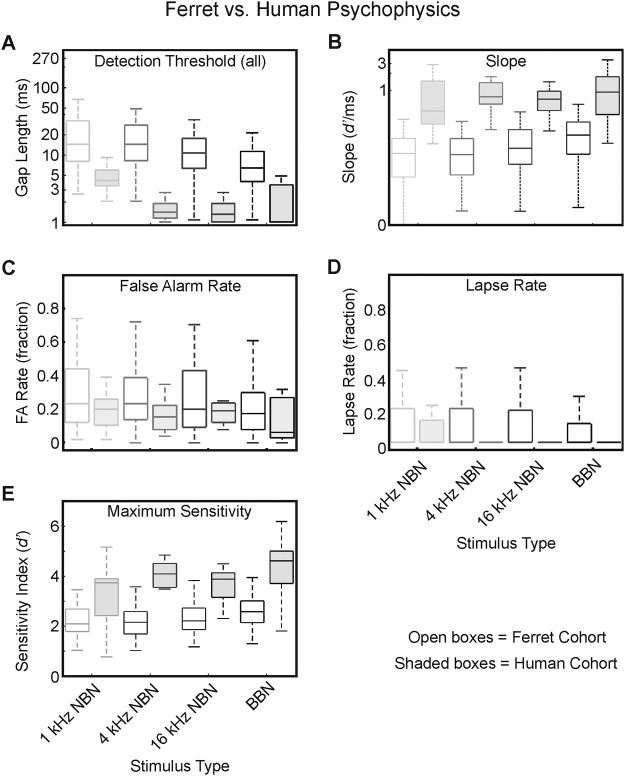
Comparison of ferret and human psychometric performance data. For each case, the open boxes represent ferret cohort data, while the shaded boxes represent human cohort data. Box plots indicate medians and interquartile ranges in each case, with whiskers illustrating maxima and minima. Box edge colors correspond to stimulus frequency: light gray = 1-kHz NBN; midgray = 4-kHz NBN; dark gray = 16-kHz NBN; black = BBN. Statistical tests in each case evaluated differences between the ferret and human cohorts, controlling for stimulus type, subject identity, and subject species. Gap-detection thresholds (A). Between-groups differences significant at *p* = .007. Psychometric function slopes at threshold (B). Between-groups differences significant at *p* < .001. False alarm rates (C). Lapse rates (D). As for Figure 5E, the absence of data for human participants was due to a lack of lapses made for the longest (easiest) stimuli. Maximum sensitivity at psychometric function asymptote (E). Between-groups differences significant at *p* < .001.

By deriving identical parameters from the gap-detection behavior of ferrets and humans, we were able to compare their performance directly (see Figure 6). We found a significant difference between ferrets and humans with respect to threshold at *d*′ = 1, *F*_(1, 438)_ = 9.27; *p* = .007 (Figure 6A). This difference in mean sensitivity may reflect a broader range of threshold values from session to session for ferrets compared with humans. We quantified this variability by calculating the standard deviation of thresholds across sessions for each animal and human subject. While within-individual variability was not affected by stimulus type (Kruskal–Wallis: ferrets, χ^2^ = 3.24, *df* = 3, 35, *p* = .36; humans, χ^2^ = 2.49, *df* = 3, 15, *p* = .48), there emerged a large difference between ferrets (mean standard deviation across sessions = 12.21 ms) and humans (mean standard deviation across sessions = 0.95 ms). There was also a highly significant main effect of species on slope at threshold, *F*_(1, 438)_ = 54.55; *p* < .001 (Figure 6B); together, these results suggest that at threshold performance, humans are capable of consistently discriminating smaller gap lengths, and finer differences in gap length, than are ferrets. There was no difference between species with respect to FA rate, *F*_(1, 438)_ = 2.22; *p* = .16 (Figure 6C), and the effects on sensitivity did not appear to result from differences of attention in our animal cohort (relative to the human group), since the lapse rate (Figure 6D) did not display significant differences as a function of subject species—lapse rate: *F*_(1, 438)_ = 3.36; *p* = .08. We did, however, find that humans showed significantly greater asymptote sensitivity than ferrets, *F*_(1, 438)_ = 61.39; *p* < .001 (Figure 6E). Notably, we did not find a significant interaction at α = .05 between stimulus type and species for any of the parameters investigated: gap-detection threshold, *F*_(3, 398)_ = 0.85, *p* = .47; slope, *F*_(3, 398)_ = 1.49, *p* = .22; FA rate, *F*_(3, 398)_ = 0.37, *p* = .78; lapse rate, *F*_(3, 398)_ = 0.21, *p* = .87; asymptote sensitivity, *F*_(3, 398)_ = 1.03, *p* = .38. Consequently, we can conclude from the effects of varying the spectral content of the stimuli that ferret gap-detection behavior resembles that exhibited by human listeners, but with diminished sensitivity.[Fig-anchor fig6]

## Discussion

This study has extended our understanding of ferret auditory behavior by providing a comprehensive investigation of gap-in-noise detection in this species. Importantly, by comparing the temporal processing abilities of each species tested under similar conditions, we have demonstrated the viability of the ferret as a useful model for investigating aspects of human auditory processing, particularly in relation to the frequency-dependent perception of temporal structure in acoustic stimuli. The establishment of this model thus paves the way for further investigation of the neural mechanisms responsible for gap-in-noise encoding, with particular translational relevance for those structures and mechanisms affected by aging and hearing loss.

By testing gap detection using broadband and different narrowband stimuli, our results demonstrate that, as in humans, ferret temporal processing depends on stimulus spectral content, with worse performance observed when low-frequency sounds were used. In the only previous report of gap detection in ferrets, Kelly et al. (1996) tested two animals on a battery of stimuli akin to the conditions used here, with one case seemingly displaying a frequency-dependent enhancement for bandpassed noise stimuli centered at frequencies above 8 kHz, whereas the other animal yielded consistent thresholds across all tested frequencies. Nevertheless, the mean thresholds reported in our study were similar to those reported by Kelly et al. (1996), despite the absence in the latter of clear evidence for stimulus frequency affecting detection sensitivity.

Our ferret cohort displayed mean thresholds as low as 11.9 ms when broadband stimuli were used, with thresholds increasing in a frequency-dependent fashion when narrowband stimuli were used. Previously reported gap-detection thresholds obtained using operant testing in smaller mammals range from 1.6 ms in mice (Radziwon et al., 2009) and rats (Syka et al., 2002) and 2.1 ms in gerbils (Wagner et al., 2003) to 3 ms in chinchillas (Giraudi et al., 1980). Our data therefore place ferrets at the upper end of the scale of gap detection in small mammals. The origin of this difference between ferrets and other animals tested is not clear, although it is notable that in individual cases, animals approached detection thresholds as low as 3 ms in single sessions, suggesting that ferrets are capable of detecting gaps to a degree comparable with other small mammals. It is also pertinent that, as indicated in Figure 2B, the reported “threshold” data in fact place only an upper bound on performance, with actual psychophysical thresholds liable to be lower. Interestingly, Syka et al. (2002) reported that gap-detection thresholds in rats were much lower at 70 dB SPL for broadband and low-frequency narrowband noise than when the stimulus level approached an audiometric boundary, where thresholds were as high as 10.6 and 31.3 ms for broadband and low-frequency noise, respectively. While not investigated in the present study, an effect of overall level on gap detection has been indicated previously in ferrets (Kelly et al., 1996) and widely explored in humans (e.g., Fitzgibbons, 1983; Penner, 1977; Plomp, 1964; Shailer & Moore, 1983). However, it is striking that the upper detection limits for rats are in line with those obtained for ferrets in the present study (BBN: 11.9; 1-kHz NBN: 24.6 ms) for gaps in noise well above the audiometric threshold, and similarly that the lower detection limits obtained in each ferret’s best sessions (Figure 2B) were much closer to those described in other species.

One possibility is that ferrets were performing suboptimally in our behavioral setup, yielding detection sensitivity values at the upper limit of a strategy that will reliably yield reward. This may have developed as a function of the positive conditioning paradigm utilized, wherein punishment of poor performance was avoided in favor of rewarding correct performance. Indeed, a previous study of detection behavior in an operantly conditioned yes/no-type task indicated that ferrets are capable of modifying their decision criterion on a trial-by-trial basis to optimize the reward probability, conditional upon the outcome of the previous trial (Alves-Pinto et al., 2012). A notable factor associated with the decision process is the FA rate which, while high in the present study, was consistent with other studies in this species (Alves-Pinto et al., 2012). This may reflect a species-related idiosyncrasy of operant behavior decision-making, given that a similar conditioning paradigm in European starlings (*Sturnus vulgaris*) yielded FA rates approaching 0.04 (Klump & Maier, 1989), and much greater maximum sensitivities at psychometric asymptote were seen in other species utilizing operant measures to obtain gap-detection thresholds (Radziwon et al., 2009; Wagner et al., 2003). However, direct comparison is problematic, since in each of those studies high FA rates were among the exclusion criteria for session analysis. Nevertheless, the fact that a proportion of animals in the present study displayed detection thresholds within approximately the same range as those previously reported in other species likely indicates a conserved central mechanism for encoding features of gap-in-noise stimuli in a behaviorally salient fashion.

It is well established that effective gap detection is contingent upon the normal operation of auditory cortical circuitry (Ison et al., 1991; Kelly et al., 1996; Threlkeld, Penley, Rosen, & Fitch, 2008; Weible et al., 2014), as well as upon the integrity of the peripheral hearing organ (Giraudi-Perry, Salvi, & Henderson, 1982; Gold, Peters, Nodal, King, & Bajo, 2013; Rybalko & Syka, 2005; Salvi & Arehole, 1985; Yin, Feng, Chen, & Wang, 2008). It has been postulated that the temporal processing underpinning perceptual gap detection is determined by the time constants of the cochlear filters in a frequency-dependent manner (Klump & Maier, 1989). Recent recordings from ferret auditory nerves have indicated the presence of an unexpectedly low proportion of fibers tuned to very low-frequency sounds, despite functionally appropriate behavioral and collicular–cortical thresholds within this range (Sumner & Palmer, 2012). These results imply that the ferret is closer in peripheral function to the chinchilla or guinea pig, rather than the cat, with which comparisons are more often drawn (Kelly, Judge, & Phillips, 1986; Phillips, Judge, & Kelly, 1988). It is interesting, therefore, that detection thresholds should be as high as they are in ferrets, particularly since the width of their auditory filters would provide shorter integration time constants, and thus ought to yield the physiological basis necessary to detect shorter gaps in noise. Although no data are available on the responses to gap-in-noise stimuli in the ferret central auditory system, a recent neurophysiological study of guinea pig inferior colliculus found mean neural gap-detection thresholds for single units to be 10.95 ms in response to broadband noise (Berger, Coomber, Wells, Wallace, & Palmer, 2014). This figure is remarkably close to our behaviorally measured thresholds (though greater than previously reported neurometric gap thresholds measured using evoked potentials in guinea pig inferior colliculus and auditory cortex; Yin et al., 2008).

It has been proposed that the well-documented reduction in gap-detection performance with age in both humans and other species (Allen, Burkard, Ison, & Walton, 2003; Barsz et al., 2002; Hamann et al., 2004; Recanzone, Engle, & Juarez-Salinas, 2011; Reed, Braida, & Zurek, 2009; Walton, Simon, & Frisina, 2002) may correlate with the progressive loss of high-frequency sensitivity (for review, see Gold & Bajo, 2014). Thus, we would predict, on the basis of the ferret’s peripheral physiology and central encoding of sound frequency (Bizley et al., 2005; Kelly & Judge, 1994; Kelly, Judge, & Phillips, 1986), that this species might be particularly susceptible to age-related impairments in the detection of temporally complex sounds. To date, aging studies on ferrets are lacking. However, on the basis of preliminary observations in partially deafened ferrets, it appears that some animals are prone to developing similar temporal processing deficits in the wake of peripheral trauma (Gold et al., 2013). Since the induction of similar lesions in this species results in central processing deficits throughout the auditory system that are typical of those seen in other species, ferrets are likely to be well suited for evaluating the effects on temporal processing of induced or acquired auditory processing deficits (Gold & Bajo, 2014; McAlpine, Martin, Mossop, & Moore, 1997; Moore, France, McAlpine, Mossop, & Versnel, 1997).

Gap detection in humans depends on sound frequency content (Fitzgibbons, 1983, 1984; Shailer & Moore, 1983), and the thresholds reported here are in line with previously published data, which showed that thresholds and asymptotic performance were both superior for broadband versus low-frequency bandpassed noise. Previous authors have reported that the lowest thresholds in humans are seen for broadband noise (Penner, 1977; Plomp, 1964) or for sounds containing high-frequency components >5 kHz (Fitzgibbons, 1983, 1984), as long as the stimulus was presented above a critical level of approximately 50 dB SPL (a factor not affecting our own data, which were collected at sound levels >70 dB SPL). These stimulus-specific effects on gap-in-noise perception may originate from the coactivation of particular auditory neurons that produce a characterizable physiological signature in the auditory cortex (Heinrich, Alain, & Schneider, 2004; Rupp, Gutschalk, Hack, & Scherg, 2002). This neural trace is likely to manifest as the putative activation of distinct perceptual channels representing acoustic information using a relative timing operation (Phillips & Hall, 2000; Phillips, Hall, Harrington, & Taylor, 1998) that occurs similarly in untrained listeners (Phillips & Smith, 2004).

Although our data do not conform with the suggestion from Eddins, Hall, and Grose (1992) that gap-detection performance does not change as a function of frequency in the low to midrange of the human audiogram, they nevertheless support the proposal of enhanced acuity as a function of stimulus bandwidth (with improved performance observed for wider-band stimuli), possibly deriving from integration of temporal information across processing channels (Shailer & Moore, 1983, 1987). Data obtained in other animal species (e.g., Ison et al., 2005; Radziwon et al., 2009) have revealed a similar stimulus dependence, as do our own results in ferrets, which also showed that stimulus frequency content affected our subjects’ sensitivity to the size of incremental changes in gap length at threshold (Figures 6A and 6B). With respect to our own data, it is interesting that the same pattern of descriptive statistics was seen in both ferrets and humans across the range of stimuli tested, in spite of clear differences in their respective overall gap-detection sensitivity.

Ferrets also show much higher thresholds than humans on a two-alternative forced-choice task, in which they were trained to label artificial vowels as high or low in pitch (Walker et al., 2009), with similar parallels between the effects of stimulus type on performance in the two species to what we observed here. Although this difference in sensitivity may reflect the complexity of the task used, one factor that is liable to have affected estimates of threshold performance is the session-to-session variability observed within each species. In the present study, we quantified this variability by calculating the standard deviation of thresholds across sessions for each animal and human subject, and in each case there was no effect of stimulus on within-individual variability. However, there was a large difference between ferrets (mean standard deviation across sessions = 12.21 ms) and humans (mean standard deviation across sessions = 0.95 ms). This would suggest that ferrets are an order of magnitude more variable in their between-session performance than humans, a factor possibly influenced by motivation. Additional explanations include issues with task design (e.g., feedback provided only to ferrets, not human subjects) and difficulty (a factor that could be addressed by implementing a method of limits in the future rather than constant stimuli as used here). There may also be fundamental interspecies differences in peripheral encoding of temporally complex acoustic stimuli as a function of differing cochlear physiology (Elliott, Stein, & Farrison, 1960) or cochlear nonlinear dynamics, which might influence gap-in-noise detection (Horwitz et al., 2011; Reed et al., 2009). In the case of the latter hypothesis, however, the ferret has been shown to display basilar membrane compressive growth at a rate comparable with other mammalian species, including guinea pigs and nonhuman primates (Sumner & Palmer, 2012).

Our key finding that both ferrets and humans display a comparable main effect of frequency content on psychometric performance supports not only the utility of our paradigm for gap-detection testing but also supports the ferret as a model species for investigating the neurobiology of temporal processing in a human-relevant manner. While it is certainly the case that the ferret’s detection thresholds are likely to be higher than those in humans and certainly are worse than those reported in other experimental mammals, as a model species the ferret possesses a number of advantages that make it suitable for continued exploration of the substrates for temporal coding. In the first case, operant conditioning as a paradigm, while influenced by attentional and motivational factors (Heffner & Heffner, 2014), provides insight into cortical processes that are critical to task performance (Kelly et al., 1996). This is in contrast to startle-based paradigms, which are predominantly mediated by subcortical networks (Yeomans & Frankland, 1995). In addition to the ferret being one of the most widely used models for the exploration of spatial hearing (King et al., 2007), in which the accurate integration of temporally precise cues is important to the localization of a sound source (Keating, Dahmen, & King, 2015; Keating et al., 2014), the appropriateness of this species for auditory research in general is enhanced by the opportunity to simultaneously investigate, over extended periods of time and within single animals, the activity of populations of neurons during behavioral tasks (Bizley, Walker, Nodal, King, & Schnupp, 2013) and the consequences of selective inhibition (Bajo et al., 2013) or ablation (Bajo, Nodal, Moore, & King, 2010) of specific neural populations.
